# Gallic Acid from *Elaeocarpus floribundus* Stem Bark: A Potent Natural Antioxidant with Enzymatic and Pharmacokinetic Validation

**DOI:** 10.3390/antiox14101161

**Published:** 2025-09-25

**Authors:** Ayorinde Victor Ogundele, Archana Moni Das, Cristian Paz

**Affiliations:** 1 Laboratory of Natural Products & Drug Discovery, Center CEBIM, Department of Basic Sciences, Faculty of Medicine, Universidad de La Frontera, Temuco 4780000, Chile; cristian.paz@ufrontera.cl; 2 Natural Products Chemistry Group, Chemical Science and Technology Division, CSIR-North East Institute of Science and Technology, Jorhat 785006, India; 3 Academy of Scientific and Innovative Research, Ghaziabad 201002, India

**Keywords:** *Elaeocarpus floribundus*, gallic acid, antioxidant behavior, ADMET, glutathione reductase, superoxide dismutase

## Abstract

The present study investigates the antioxidant potential of the stem bark of *Elaeocarpus floribundus* Blume through an integrated approach involving phytochemical isolation, in vitro radical scavenging assays, ADMET-based safety profiling, and molecular docking. Bioassay-guided fractionation of the ethanolic extract into hexane, chloroform, and ethyl acetate fractions revealed the ethyl acetate fraction to possess the highest antioxidant activity, with an IC_50_ value of 6.19 μg/mL in the DPPH assay, surpassing that of ascorbic acid (IC_50_ = 9.74 μg/mL). Subsequent isolation and characterization from the ethyl acetate fraction of the stem bark yielded five known compounds from this plant part for the first time, including gallic acid and epigallocatechin gallate. Both compounds showed potent radical scavenging activity in vitro. Among these, gallic acid exhibited superior pharmacokinetic and safety profiles based on in silico ADMET predictions, no Lipinski’s rule violations, and no predicted toxicity. Molecular docking studies showed that gallic acid had high binding affinities for glutathione reductase (GR) and superoxide dismutase (SOD), exceeding those of their reference inhibitors. A docking analysis further revealed stable interactions with catalytically relevant residues, suggesting a stabilizing modulatory effect on redox homeostasis. These findings identify *E. floribundus* stem bark as a novel source of antioxidant compounds and highlight gallic acid as a promising therapeutic candidate for oxidative stress-related disorders.

## 1. Introduction

Oxidative stress, characterized by an imbalance between reactive oxygen species (ROS) and the body’s antioxidant defenses, plays a central role in the pathogenesis of numerous chronic and degenerative diseases, including neurodegenerative disorders, diabetes, cardiovascular diseases, and cancer [1]. Naturally occurring antioxidants, particularly phenolic compounds from medicinal plants, have gained significant attention due to their ability to scavenge free radicals, modulate enzymatic activities, and offer protective effects with minimal toxicity [2].

The genus *Elaeocarpus*, comprising over 350 species widely distributed across tropical and subtropical regions, has been a valuable source of diverse secondary metabolites with promising biological activities [3]. *Elaeocarpus floribundus* Blume, commonly known as Indian olive and locally referred to as “jolphai,” is traditionally used in various regions of the world for the treatment of ulcers, dysentery, diabetes, and inflammatory conditions such as rheumatism and gingivitis [4,5]. Ethnopharmacological claims are supported by studies demonstrating the antimicrobial, antidiabetic, and antioxidant effects of its fruits [6], leaves [7], and seeds [8]. Previous investigations have identified several bioactive constituents, including flavonoids, triterpenoids, sterols, and phenolic acids, from different parts of the plant [9,10]. Notably, gallic acid, a well-characterized antioxidant, was recently isolated from the seeds of *E. floribundus* and shown to possess potent free radical scavenging and antimicrobial activities [8].

Despite these findings, the stem bark of *E. floribundus* has remained underexplored in terms of its phytochemical composition and biological potential. Previous research on the stem bark extract revealed considerable free radical scavenging potential; however, the investigation did not identify these bioactive constituents nor provide mechanistic insights into their mode of action [11]. Given the traditional use of the bark decoctions in inflammatory conditions and the established correlation between oxidative stress and inflammation, exploring the antioxidant profile of the stem bark is both pharmacologically and therapeutically relevant.

In this study, we investigated the antioxidant potential of the stem bark of *E. floribundus*, aiming to isolate and characterize its bioactive constituents, assess their radical scavenging capacity, and evaluate their drug-likeness and safety profile through in silico ADMET analysis. Furthermore, to elucidate the potential mechanism of action, molecular docking studies were conducted, targeting key antioxidant enzymes: glutathione reductase (GR) and superoxide dismutase (SOD). This integrated experimental and computational approach provides valuable insights into the pharmacological relevance of *E. floribundus* bark as a source of therapeutic antioxidants.

## 2. Materials and Methods

### 2.1. General Information

Nuclear magnetic resonance (NMR) spectra (^1^H and ^13^C NMR) were recorded in deuterated solvents using JEOL ECZ400R (Tokyo, Japan, 400 MHz) and Bruker Avance III FT (Billerica, MA, USA, 500 MHz) spectrometers, with tetramethylsilane (TMS) used as the internal standard. Chemical shifts (δ) are reported in ppm, and coupling constants (J) are given in hertz (Hz). Infrared (IR) spectra were obtained using a Perkin Elmer FT-IR 2000 spectrometer (Waltham, MA, USA). Optical rotation measurements were performed in methanol (MeOH) using an AUTOPOL I polarimeter (Cape Town, South Africa). Column chromatography was carried out on silica gel (60–120, 100–200, and 230–400 mesh) using solvent mixtures of increasing polarity as eluents. The purity of the eluates was monitored on pre-coated silica gel 60 F_254_ TLC plates (Merck, Bangalore, India), visualized with the aid of UV light (254 nm), exposure to iodine vapor, and spraying with p-anisaldehyde stain reagent followed by heating at 70 °C. Solvents and fractions were concentrated using a rotary evaporator at 45 °C. Solvents used in this study were distilled before use and dried over appropriate drying agents. High-resolution mass spectrometry (HRMS) was conducted using a Waters Xevo XS QTOF mass spectrometer, Bangalore, India.

### 2.2. Plant Material, Extraction, and Isolation

The stem bark of *Elaeocarpus floribundus* was collected in March 2019 from a mature tree on the campus of CSIR–North East Institute of Science and Technology, Jorhat, India (26°44′08.3″ N, 94°09′38.4″ E), during a period that coincided with favorable weather conditions for harvesting and drying. A voucher specimen (NEIST/1893) was deposited at the institute’s herbarium. The powdered bark (2.1 kg) was subjected to three 72 h maceration processes with ethanol (5 L) at room temperature (~25 °C). The extracts were pooled, filtered through Whatman No. 1 filter paper, and concentrated under reduced pressure using a rotary evaporator at 45 °C, yielding a blood-red flaky crude extract (135 g). The crude extract was subsequently partitioned between distilled water and solvents of increasing polarity (hexane, chloroform, and ethyl acetate), affording three fractions: hexane (HB, 3.0 g), chloroform (CB, 3.1 g), and ethyl acetate (EB, 4.0 g). The antioxidant potential of the crude extract and fractions was evaluated using the DPPH radical scavenging assay, with the ethyl acetate fraction exhibiting the highest activity. This fraction was subjected to silica gel column chromatography (100–200 mesh) and eluted with solvent gradients ranging from 100% hexane to 100% ethyl acetate (100:0 to 0:100, *v*/*v*). Based on TLC profiling, eluates were combined into six major fractions (EBA–EBF). Further purification of the EBB fraction (256 mg) using silica gel chromatography (hexane/chloroform gradient) yielded pentadecanoic acid (**1**) (14 mg). β-Sitosterol (**2**) (451 mg) precipitated from the EBC fraction and was purified by acetone washing. The EBC fraction (250 mg) was further purified via silica gel chromatography (hexane/chloroform gradient) to afford 2,4-di-tert-butylphenol (**3**) (16 mg). Purification of the EBD fraction (971 mg) via silica gel chromatography (chloroform/methanol gradient) yielded gallic acid (**4**) (205 mg) and epigallocatechin gallate (**5**) (388 mg).

### 2.3. Quantification of Total Phenolic and Total Flavonoid Contents

The total reducing capacity, commonly referred to as the total phenolic content (TPC) of the ethanolic extract and its fractions, was estimated using the Folin–Ciocalteu (F-C) reagent following standard protocols [12]. It is important to note that the F–C assay responds to a range of reducing substances, including but not limited to phenolic compounds. The TPC was quantified using a gallic acid calibration curve, and the results are expressed as milligrams of gallic acid equivalent (GAE) per milligram of dry weight (mg GAE/g d.w) of the extract or fractions. The TFC was assessed using a colorimetric aluminum chloride assay following a previously described method [13], which primarily detects flavonols and flavones based on their ability to form stable complexes with AlCl_3_. The TFC was determined using a rutin calibration curve and expressed as milligrams of rutin equivalent (RE) per milligram of dry weight (mg RE/g d.w) of the extract or fractions. Due to structural specificity, this method may not capture all subclasses of flavonoids uniformly. All measurements were performed in triplicate.

### 2.4. Radical Scavenging Potential Towards DPPH Free Radicals

The antioxidant potential of the stem bark as a cost-effective and natural alternative to synthetic antioxidants was evaluated using the DPPH free radical scavenging assay following the method detailed in [14]. Different concentrations (100 μg/mL to 5 mg/mL) of the samples were combined with an equal volume of 0.3 mM DPPH solution prepared in methanol. The mixture was thoroughly shaken and left to incubate in the dark at room temperature for 30 min. Absorbance readings were then taken at 517 nm using a spectrophotometer. Ascorbic acid served as the reference antioxidant, while a 0.3 mM methanol solution of DPPH acted as the control. The percentage inhibition was calculated using the following formula:

% inhibition = [(Absorbance of control − Absorbance of sample)/Absorbance of control] × 100.

### 2.5. Computational Toxicity Studies

The ADMET (absorption, distribution, metabolism, excretion, and toxicity) properties of the isolated compounds were predicted using in silico tools. The *Swiss*ADME server (http://www.swissadme.ch/, accessed on 10 December 2024) was employed to evaluate pharmacokinetic parameters, including absorption, distribution, and metabolism [15]. The toxicity profile of the compounds was further assessed using the *pk*CSM online tools (https://biosig.lab.uq.edu.au/pkcsm/prediction, accessed on 10 December 2024), which provide toxicity class predictions based on chemical structure [16]. These computational analyses were performed to predict the drug-likeness and potential toxicological risks associated with the isolated bioactive compounds.

### 2.6. Molecular Docking Studies

For molecular docking studies, the 3D structure of gallic acid and the standard were obtained from the PubChem database (https://pubchem.ncbi.nlm.nih.gov/, accessed on 02 December 2024) in SDF format and converted to PDB format using Open Babel software 2.4.1 [17]. Gallic acid was selected for molecular docking against GR and SOD based on its favorable pharmacokinetic profile, exhibiting no violations of Lipinski’s rules, no predicted toxicity risks, and superior in vitro antioxidant activity compared to the reference compound, ascorbic acid. The co-crystalized ajoene inhibitor was used as the reference ligand for GR, while quercetin served as the reference ligand for SOD [18].

The crystal structures of GR (PDB ID: 1BWC) and SOD (PDB ID: 2C9V) were retrieved from the Protein Data Bank (http://www.rcsb.org/pdb, accessed on 20 December 2024). The GR structure was co-crystallized with the ligand ajoene, which served as the reference inhibitor. This enabled targeted docking at the inhibitor binding site and provided insights into the key amino acid residues involved in GR inhibition. For SOD, the grid box center and size coordinates were obtained from a previous study [18], allowing for targeted docking at its active site.

The crystallized enzyme structures were processed using UCSF ChimeraX v1.8. Before analysis, water molecules and co-crystallized ligands were eliminated, followed by the addition of polar hydrogen atoms to account for the protonation states of ionizable amino acid residues under physiological conditions. After the preparation of the ligands and crystallized enzymes, molecular docking was performed using AutoDock Vina v1.1.2 [19]. The resulting docking poses were analyzed and visualized using BIOVIA Discovery Studio v21.1.0.20298.

### 2.7. Statistical Analysis

All experimental procedures were performed in triplicate using three independently prepared samples. Data are presented as mean ± standard deviation (SD). Statistical significance between groups was assessed using a one-way analysis of variance (ANOVA) followed by Tukey’s post hoc test, where applicable. A *p*-value of <0.05 was considered statistically significant. All analyses were performed using Microsoft Excel 2016 with the Real Statistics Resource Pack add-in.

## 3. Results

### 3.1. Isolation of Compounds

**Figure 1:**
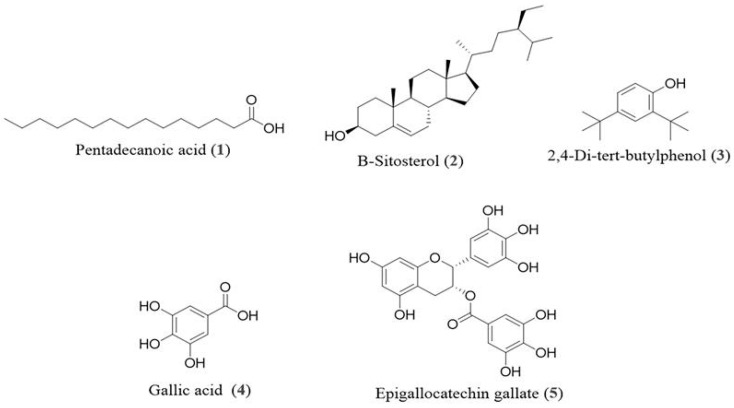
Chemical structures of pentadecanoic acid (**1**), β-Sitosterol (**2**), 2,4-di-tert-butylphenol (**3**), gallic acid (**4**), and epigallocatechin gallate (**5**) from stem bark of *E. floribundus*.

The structures of the compounds (**1**–**5**) were identified through a comparison of their MS and NMR data (Figures S1–S5) with those reported previously, including pentadecanoic acid (**1**) [20], β-Sitosterol (**2**) [21], 2,4-di-tert-butylphenol (**3**) [22], gallic acid (**4**) [23], and epigallocatechin gallate (**5**) [24], and the structures are shown in Figure 1.

### 3.2. Quantification of TPC, TFC, and DPPH Scavenging Activity of Extract and Fractions of E. floribundus

The stem bark of *Elaeocarpus floribundus* underwent ethanol extraction and was further fractionated using solvents of increasing polarity: hexane, chloroform, and ethyl acetate. The total reducing capacity (commonly interpreted as the total phenolic content, TPC), total flavonoid content (TFC), and 2,2-diphenyl-1-picrylhydrazyl (DPPH) radical scavenging activity of the crude extract and its fractions were assessed and are summarized in Table 1. The crude extract displayed the highest TPC (65.46 ± 0.31 mg/g), followed by the ethyl acetate fraction with a value of 58.71 ± 2.05 mg/g. In contrast, the chloroform and hexane fractions had significantly lower values (*p* < 0.05). A similar trend was observed for the total flavonoid content (TFC) and DPPH scavenging activity, where the ethyl acetate fraction showed significantly stronger antioxidant activity (IC_50_ = 6.19 ± 0.03 μg/mL) compared to the other fractions (*p* < 0.05). No statistically significant difference was observed between the TPC of the ethanol extract and the ethyl acetate fraction (*p* > 0.05), suggesting that both are comparably rich in phenolic compounds.

### 3.3. DPPH Antioxidant Inhibitory Activity of Isolated Components

To further investigate the potential applications of the isolated main compounds (Figure 1), pentadecanoic acid (**1**), β-Sitosterol (**2**), 2,4-di-tert-butylphenol (**3**), gallic acid (**4**), and epigallocatechin gallate (**5**) were evaluated for their radical scavenging activity. The isolated compounds demonstrated dose-dependent DPPH radical scavenging activity, as shown in Table 2. Notably, epigallocatechin gallate (**5**) and gallic acid (**4**) demonstrated significantly higher antioxidant activity, with IC_50_ values of 8.05 ± 0.17 μM and 16.04 ± 0.07 μM, respectively, which were significantly different (*p* < 0.05) from the weaker or inactive compounds. Their activities surpassed the standard, ascorbic acid (55.29 ± 0.22 μM), confirming their relevance to the extract’s potency. Compounds **1** and **2** showed negligible activity (>500 μM), while compound **3** exhibited moderate scavenging potential (IC_50_ = 125.05 ± 0.20 μM). Statistical analysis using a one-way ANOVA followed by Tukey’s post hoc test confirmed that the differences among compounds were statistically significant (*p* < 0.05). These findings suggest that compounds **4** and **5** are the primary contributors to the strong antioxidant properties of the ethyl acetate fraction.

### 3.4. Prediction of Pharmacokinetic and Toxicological Properties

**Figure 2:**
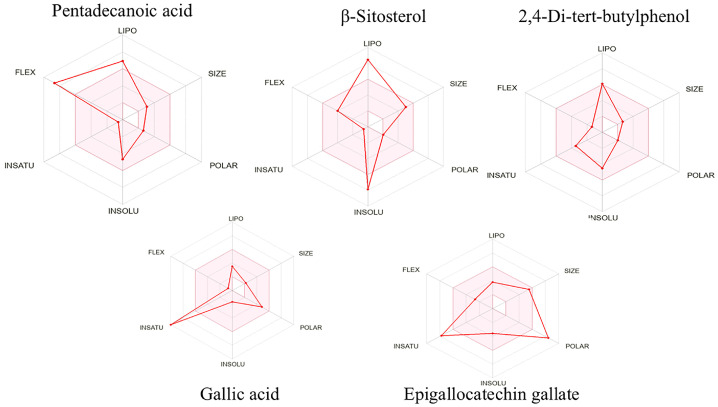
Bioavailability radars of isolated compounds.

The pharmacokinetic behavior and toxicological potential of the phytochemical constituents isolated from *E. floribundus* stem bark were evaluated using the *Swiss*ADME and *pk*CSM web tools. The results are summarized in Table 3, while the bioavailability radars in Figure 2 provide a graphical comparison of their physicochemical profiles. For these bioactive compounds to qualify as promising oral therapeutics targeting antioxidant pathways, they must satisfy two critical criteria: First, they should demonstrate favorable drug-likeness by exhibiting no more than one violation of Lipinski’s Rule of Five. Second, comprehensive toxicity screening should confirm that they pose no significant risks, showing no indications of mutagenic potential, carcinogenicity, tissue irritation, or adverse effects on reproductive function [25]. This two-tiered evaluation approach helps identify compounds with favorable bioavailability and safety profiles. Among the five isolated phytochemicals, only epigallocatechin gallate (**5**) exhibited multiple violations of Lipinski’s Rule of Five, with all four showing either complete compliance or only one violation of these established pharmacokinetic guidelines.

Gallic acid, which complied with Lipinski’s rules, exhibited no predicted toxicity risk, and demonstrated superior in vitro radical scavenging activity compared to the standard, was selected as a potential inhibitor of glutathione reductase (GR) and superoxide dismutase (SOD) for targeting antioxidant pathways. It was therefore evaluated through docking analysis to assess its inhibitory performance in comparison with the reference inhibitors: ajoene for GR and quercetin for SOD. Moreover, ADMET parameters revealed favorable drug-like properties, supporting its potential therapeutic applications.

### 3.5. Determination of Antioxidant Activity Through Molecular Docking

Given the superior in vitro antioxidant activity of gallic acid compared to the standard ascorbic acid, along with its excellent drug-likeness profile and absence of predicted toxicity as revealed by ADMET analysis, molecular docking was performed to further explore its potential mechanism of action. GR and SOD, two key enzymes involved in the antioxidant defense system, were selected as molecular targets. GR is a key enzyme that maintains the intracellular pool of reduced glutathione (GSH), essential for neutralizing ROS and preserving redox homeostasis. The inhibition or dysfunction of GR can impair glutathione recycling, leading to increased oxidative stress and cellular damage [26]. SOD, on the other hand, catalyzes the dismutation of the superoxide radical into hydrogen peroxide and molecular oxygen, thereby serving as the first line of enzymatic defense against ROS [27]. Together, GR and SOD form a crucial part of the antioxidant machinery, and their modulation can significantly influence oxidative stress-related pathways. The current therapeutic landscape for targeting antioxidant pathways is constrained by efficacy challenges and toxicity concerns, driving the search for improved compounds from natural origins that potentially exhibit fewer detrimental side effects. Hence, docking studies were conducted to assess the binding affinity and interaction patterns of gallic acid with the active sites of these enzymes in comparison with their respective reference inhibitors.

**Figure 3:**
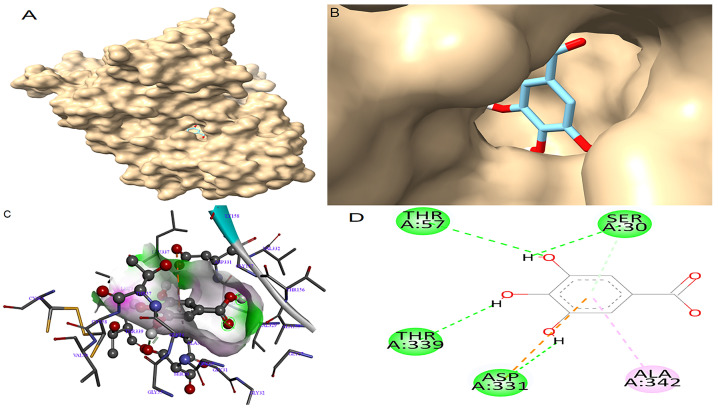
Molecular docking between gallic acid and GR. (**A**) The adopted molecular geometry of gallic acid in the catalytic pocket of the GR enzyme; (**B**) a zoomed view of the geometry adopted by gallic acid in the catalytic pocket of GR; (**C**) an analysis of the hydrogen bonds of gallic acid–GR complex; (**D**) a map of predominant interactions of the molecular docking of gallic acid and GR.

Molecular docking was performed to generate the ten most stable conformations of gallic acid from which the conformation with the highest binding affinity was selected for analysis. The docking results are summarized in Table 4. For GR, gallic acid exhibited a notable binding affinity of −10.04 kcal/mol, surpassing that of the reference inhibitor ajoene, which recorded a binding affinity of −8.98 kcal/mol. The binding pose of gallic acid within the catalytic pocket of GR and its spatial orientation in the active site are illustrated in Figure 3A and Figure 3B, respectively. The detailed interaction map is shown in Figure 3C. Gallic acid formed four conventional hydrogen bonds with key residues in the GR active site: one between the hydroxyl group on its phenyl ring and the side chain of Ser30; another between a separate hydroxyl group and Thr57; a third between a hydroxyl group and Thr339; and a fourth involving a hydrogen bond between a hydroxyl group and the carboxylate side chain of Asp331. In addition to hydrogen bonding, gallic acid established a π-alkyl interaction with Ala342 as well as a π-anion interaction between its aromatic ring and the negatively charged residue Asp331.

**Figure 4:**
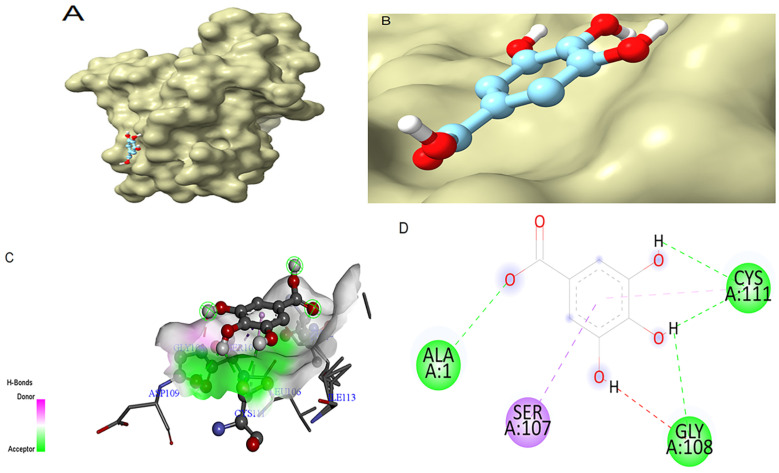
Molecular docking between gallic acid and SOD. (**A**) The adopted molecular geometry of gallic acid in the catalytic pocket of the SOD enzyme; (**B**) a zoomed view of the geometry adopted by gallic acid in the catalytic pocket of SOD; (**C**) an analysis of the hydrogen bonds of gallic acid–SOD complex; (**D**) a map of predominant interactions of the molecular docking of gallic acid and SOD.

In the case of SOD, gallic acid showed a theoretical binding energy of −9.84 kcal/mol, while the standard quercetin displayed a binding score of −7.54 kcal/mol. The binding pose of gallic acid within the catalytic pocket of SOD and its spatial orientation in the active site are illustrated in Figure 4A and Figure 4B, respectively. The detailed interaction map is shown in Figure 4C,D. Gallic acid interacts with SOD, forming H-bonds at the receptor site interacting region involving residues Cys111, Gly108, and Ala1. SOD residue Ser107 was involved in the π-σ interaction.

## 4. Discussion

This study provides a detailed evaluation of the antioxidant potential of *Elaeocarpus floribundus* stem bark, employing a multidisciplinary approach involving extraction, phytochemical isolation, in vitro antioxidant assays, pharmacokinetic and toxicity profiling, and molecular docking studies. The results highlight the plant’s potential as a valuable source of natural antioxidants, particularly phenolic compounds.

The ethanol extract and its solvent-partitioned fractions showed varying levels of TPC, TFC, and radical scavenging activity. The crude extract exhibited the highest TPC (65.46 mg GAE/g) and TFC (20.82 mg RE/g). Among the fractions, the ethyl acetate fraction stood out, not only for its high TPC (58.71 mg GAE/g) but also for its superior DPPH scavenging activity (IC_50_ = 6.19 μg/mL), outperforming the standard antioxidant ascorbic acid (IC_50_ = 9.74 μg/mL).

The antioxidant activity and polyphenolic content observed in this study are consistent with, and in some cases superior to, previous reports on various parts of the same species. For instance, Rahayu Utami et al. (2013) reported a significantly higher TPC of 161.5 ± 24.81 mg GAE/g DW for the methanolic extract of the stem bark compared to the value of 65.46 mg GAE/g extract in our study [11]. This difference may result from variations in solvent polarity and extraction efficiency, expression units (extract weight vs. dry weight), and other biological and environmental factors such as geographical origin, plant maturity, and the seasonal timing of collection, all of which are known to affect secondary metabolite composition. In terms of antioxidant activity, their reported DPPH IC_50_ value of 7.36 ± 0.01 μg/mL for the methanol stem bark extract closely matches the IC_50_ value of 6.19 μg/mL found in our ethyl acetate fraction, supporting the reproducibility of strong antioxidant potential in this plant part. Flavonoid content comparisons further support the relevance of our findings. Utami et al. (2023) reported a TFC of 60.59 ± 0.53 mg QE/g extract in ethyl acetate leaf extracts, whereas our ethyl acetate bark fraction exhibited a significantly lower TFC of 8.05 mg RE/g [28]. Our previous work on the fruit of the same plant showed a TPC of 0.5 mg GAE/mg and a TFC of 0.2 mg RE/mg in the crude extract, with significantly lower levels detected in the partitioned fractions [6]. In the current study, the TPC and TFC of the ethyl acetate bark fraction were notably higher (58.71 mg GAE/g and 8.05 mg RE/g, respectively), further establishing the bark as a richer source of antioxidant-relevant compounds. Similarly, Bijayanta and Shyamapada (2017) reported a TPC range of 0.087 to 1.39 mg/g for the ethanolic fruit extract, significantly lower than the values observed in this study for the bark extract and its fractions [29]. Likewise, the DPPH radical scavenging activity of the ethyl acetate fraction (IC_50_ = 6.19 μg/mL) was more potent than that of the fruit extract in our previous work, which exhibited an IC_50_ value of 2.05 mg/mL [6]. Although the seed extract showed stronger antioxidant activity (IC_50_ = 4.4 μg/mL), as reported in our previous study [8], the bark remains a valuable source due to its combination of significant in vitro antioxidant potential, high phenolic content, and superior pharmacokinetic and toxicity profiles demonstrated in this study. Collectively, these comparisons emphasize the antioxidant potential of the stem bark, particularly the ethyl acetate fraction, and validate the selection of this plant part for further phytochemical investigation.

It is worth emphasizing that the Folin–Ciocalteu assay used for estimating the TPC is not specific to phenolic compounds but rather reflects the total reducing capacity of a sample. This includes contributions from non-phenolic lowering agents such as ascorbic acid, certain amino acids, and reducing sugars [30,31]. As such, while high TPC values in the crude and ethyl acetate fractions likely reflect an enriched polyphenolic content, the low TPC recorded in the chloroform fraction may not necessarily indicate the absence of antioxidant-relevant compounds. Instead, it may reflect both the reduced polarity of the solvent, which selectively extracts fewer phenolic compounds and the limitations of the F–C assay itself in accurately distinguishing phenolics from other reductants. Additionally, the aluminum chloride assay used to estimate the total flavonoid content selectively detects flavonoids with certain structural features, such as flavonols and flavones. It may underestimate the presence of other flavonoid subclasses [32].

The subsequent isolation and characterization of compounds from the ethyl acetate fraction led to the identification of five compounds, among which gallic acid (**4**) and epigallocatechin gallate (**5**) exhibited potent radical scavenging activity in the DPPH assay (IC_50_ = 16.04 μM and 8.05 μM, respectively), exceeding that of ascorbic acid (IC_50_ = 55.29 μM). These results suggest that the antioxidant activity of the ethyl acetate fraction is largely attributed to its phenolic constituents, which are known for their electron-donating capacity and ability to stabilize free radicals [33].

ADMET predictions further supported the suitability of gallic acid for therapeutic development. It exhibited no violations of Lipinski’s Rule of Five, high gastrointestinal absorption, and no predicted toxicity. The pharmacokinetic simulation results obtained for gallic acid in this study, indicating high gastrointestinal absorption, the absence of hepatotoxicity and mutagenicity, and no violations of Lipinski’s rules, are consistent with existing experimental data in the literature. Gallic acid has been previously reported to exhibit favorable oral bioavailability and rapid intestinal absorption in animal studies, supporting its potential for systemic antioxidant effects [34,35]. Toxicological studies have shown that gallic acid has a high safety margin, with no observed adverse effects in rodents even at relatively high doses [36]. These findings corroborate the outcomes of our in silico analysis and support the suitability of gallic acid as a safe and effective antioxidant agent with translational relevance. In contrast, although epigallocatechin gallate showed strong in vitro activity, its poor drug-likeness profile due to multiple Lipinski violations made it less ideal for further docking analysis.

To investigate the mechanism behind gallic acid’s antioxidant action, molecular docking was performed against GR and SOD, key enzymes involved in intracellular redox regulation. GR regenerates reduced GSH [37], while SOD catalyzes the dismutation of superoxide radicals [38]. Both enzymes are vital for neutralizing ROS and preventing oxidative damage.

Docking poses were analyzed and compared to their respective antioxidant standards. In this study, gallic acid docked very well compared to the standards. The docking results show that gallic acid had strong binding affinities for both enzymes (−10.04 kcal/mol for GR and −9.84 kcal/mol for SOD), surpassing the reference inhibitors ajoene (−8.98 kcal/mol) and quercetin (−7.54 kcal/mol), respectively. Gallic acid formed stable interactions through multiple hydrogen bonds and π interactions with catalytically important residues, such as Thr57 and Thr339 in GR [39] and Cys111, Ala1, and Gly108 in SOD [18]. These interactions suggest that gallic acid may enhance or stabilize antioxidant enzyme function, and this stability is crucial for its potential inhibitory activity, thus contributing to its potent in vitro effects [40].

While five major compounds were isolated and structurally elucidated from the ethyl acetate fraction, their quantities within the fraction were not determined in this study. Nonetheless, based on their high radical scavenging capacity, particularly gallic acid and epigallocatechin gallate, and their consistent presence in bioactive polar fractions across similar studies [41,42], it is reasonable to infer that these compounds play a central role in the observed activity. However, the ethyl acetate fraction is likely to contain other polyphenolic constituents not captured in this study. High molecular weight tannins, polymeric flavonoids, and phenolic glycosides, commonly found in *Elaeocarpus* species and other medicinal plants, could also contribute significantly to the antioxidant potential [11,43]. Previous phytochemical studies on *E. floribundus* and related species have reported the presence of ellagitannins, proanthocyanidins, and other complex phenolic structures [44,45], which were not isolated here but may be present in minor or less extractable quantities. Future studies incorporating LC-MS or quantitative HPLC profiling are necessary to fully characterize the composition and relative abundance of antioxidant-active constituents in the extract.

Collectively, these findings establish *Elaeocarpus floribundus* Blume stem bark as a promising source of antioxidant compounds, with gallic acid emerging as a particularly potent, pharmacologically safe, and mechanistically validated lead compound. Although the results are promising, the lack of in vivo or ex vivo validation limits the extent to which the antioxidant effects of gallic acid can be extrapolated to physiological contexts. Therefore, further biological studies are necessary to confirm its therapeutic potential.

## 5. Conclusions

This study offers detailed insights into the antioxidant potential of the stem bark of *Elaeocarpus floribundus* Blume. The ethyl acetate fraction demonstrated remarkable radical scavenging activity, prompting the isolation of key bioactive constituents, with gallic acid exhibiting the highest antioxidant activity among them. Gallic acid exhibited superior in vitro antioxidant activity compared to ascorbic acid and met essential pharmacokinetic and safety criteria in silico based on ADMET predictions. Molecular docking further supported its high binding affinity and predicted stable interactions with GR and SOD, two central enzymes in cellular antioxidant defense. These findings suggest that *E. floribundus* stem bark is a valuable source of antioxidant compounds and highlight gallic acid as a promising lead compound for the development of therapeutic agents targeting oxidative stress-related diseases.

Beyond therapeutic prospects, the demonstrated antioxidant potential and safety profile of the extract and its constituents suggest possible applications in health protection and industry. These include incorporation as natural antioxidants in functional foods, nutraceuticals, and cosmeceutical products or use as natural preservatives in food and pharmaceutical formulations. While this study employed the DPPH assay, which primarily reflects hydrogen atom transfer (HAT)-based mechanisms, future studies should incorporate complementary assays such as FRAP, ORAC, CUPRAC, and ABTS to enable a broader evaluation of antioxidant capacity. Future studies involving in vivo validation and pharmacodynamic assessments are warranted to further explore its therapeutic potential against oxidative stress-related conditions. It should be noted that the present findings are based on material collected from a specific location and time point; future research should investigate seasonal and geographical influences on phytochemical composition and bioactivity.

## Figures and Tables

**Table 1:** Total phenolic content (TPC), total flavonoid content (TFC), and DPPH radical scavenging activity (expressed as IC_50_) of crude extract and solvent fractions of *E. floribundus* stem bark (mean ± standard deviation).

Samples	TPC (mg GAE/g) ^a^	TFC (mg RE/g) ^b^	DPPH IC_50_ (μg/mL) ^c^
Ethanol extract	65.46 ± 0.31 ^a^	20.82 ± 0.58 ^a^	15.53 ± 0.48 ^b^
Ethyl acetate fraction	58.71 ± 2.05 ^a^	8.05 ± 2.13 ^b^	6.19 ± 0.03 ^a^
Chloroform fraction	18.21 ± 2.35 ^b^	<1 ^c^	792.81 ± 0.09 ^c^
Hexane fraction	<1 ^c^	<1 ^c^	1050 ± 0.10 ^c^
Ascorbic acid	-	-	9.74 ± 0.22 ^a^

Values are expressed as mean ± SD (*n* = 3). Values with different superscript letters in same column are significantly different at *p* < 0.05 (one-way ANOVA followed by Tukey’s post hoc test). ^a^ Total phenolic content is expressed in milligrams of gallic acid equivalents (mg GAE/g sample). ^b^ Total flavonoid content is expressed in milligrams of rutin equivalents (mg RE/g sample). ^c^ IC_50_ values represent concentration of extract or compound required to inhibit 50% of DPPH radicals. Lower IC_50_ indicates higher antioxidant activity.

**Table 2:** Antiradical activity towards DPPH of isolated compounds.

Compounds	IC_50_ (μM) *
	DPPH
Pentadecanoic acid (**1**)	>500 ^b^
β-sitosterol (**2**)	>500 ^b^
2,4-di-tert-butylphenol (**3**)	125.05 ± 0.20 ^b^
Gallic acid (**4**)	16.04 ± 0.07 ^a^
Epigallocatechin gallate (**5**)	8.05 ± 0.17 ^a^
Ascorbic acid	55.29 ± 0.22 ^b^

IC_50_ values are expressed as mean ± SEM (*n* = 3). * Lower IC_50_ values indicate higher antioxidant activity. Different superscript letters in same column indicate statistically significant differences (*p* < 0.05) as determined by one-way ANOVA followed by Tukey’s post hoc test.

**Table 3:** Predicted ADMET properties and drug-likeness profiles of isolated compounds.

Pharmacokinetics/Drug-Likeness					
Entry	Pentadecanoic Acid	β-Sitosterol	2,4-di-tert- butylphenol	Gallic Acid	Epigallocatechin Gallate
MW	242.40	414.71	206.32	170.12	458.37
#Rotatable bonds	13	6	2	1	4
#H-bond acceptors	2	1	1	5	11
#H-bond donors	1	1	1	4	8
TPSA	37.30	20.23	20.23	97.99	197.37
Consensus log Po/w	4.84	7.24	3.99	0.21	0.95
ESOL class	−4.66	−7.90	−4.55	−1.64	−3.56
GI absorption	High	Low	High	High	Low
BBB permeant	Yes	No	Yes	No	No
Pgp substrate	No	No	No	No	No
CYP1A2 inhibitor	Yes	No	No	No	No
CYP2C19 inhibitor	No	No	No	No	No
CYP2C9 inhibitor	Yes	No	No	No	No
CYP2D6 inhibitor	No	No	Yes	No	No
CYP3A4 inhibitor	No	No	No	Yes	No
log Kp (cm/s)	−3.07	−2.20	−3.87	−6.84	−8.27
Lipinski #violations	0	1	0	0	2
Synthetic accessibility	2.20	6.30	1.43	1.22	4.20
			Toxicity		
AMES toxicity	No	No	No	No	No
Hepatotoxicity	No	No	No	No	No
hERG I/II inhibitors	No	No/Yes	No	No	No/Yes
Skin sensitization	Yes	No	Yes	No	No

**Table 4:** Docking analysis of gallic acid against two antioxidant enzymes compared to their reference inhibitors.

Compounds	Docking Score (-) (kcal/mol)	
	PDB ID: 1BWC (Glutathione Reductase)	PDB ID: 2C9V (Superoxide Dismutase)
Gallic acid	10.04	9.84
Quercetin	-	7.54
Ajoene	8.98	-

## Data Availability

The samples and any additional research data are available from the authors upon request.

## Supplementary Materials

The following supporting information can be downloaded at https://www.mdpi.com/article/10.3390/antiox14101161/s1. Copies of NMR and HRMS spectra of the isolated compounds.
