# Supplementary material for: Gallic Acid from Elaeocarpus floribundus Stem Bark: A Potent Natural Antioxidant with Enzymatic and Pharmacokinetic Validation
Source: Antioxidants (Basel). 2025 Sep 25;14(10):1161. doi: 10.3390/antiox14101161 (PMC12561868; doi:10.3390/antiox14101161)
Supplement: Supplementary file 1 [file antioxidants-14-01161-s001.zip › antioxidants-3689897-supplementary.pdf]

Supplementary data

Gallic acid from *Elaeocarpus floribundus* stem bark: A potent natural antioxidant with enzymatic and pharmacokinetic validation

Ayorinde Victor Ogundele[1*], Archana Moni Das[2, 3] and Cristian Paz[1],

[1]Laboratory of Natural Products & Drug Discovery, Center CEBIM, Department of Basic Sciences, Faculty of Medicine, Universidad de La Frontera, Temuco 4780000, Chile; vicshow2001@gmail.com, cristian.paz@ufrontera.cl
[2]Natural Products Chemistry Group, Chemical Science and Technology Division, CSIR-North East Institute of Science and Technology, Jorhat, Assam 785006, India
[3]Academy of Scientific and Innovative Research, Ghaziabad 201002, India; archanads2222@gmail.com
*Correspondence: vicshow2001@gmail.com ;

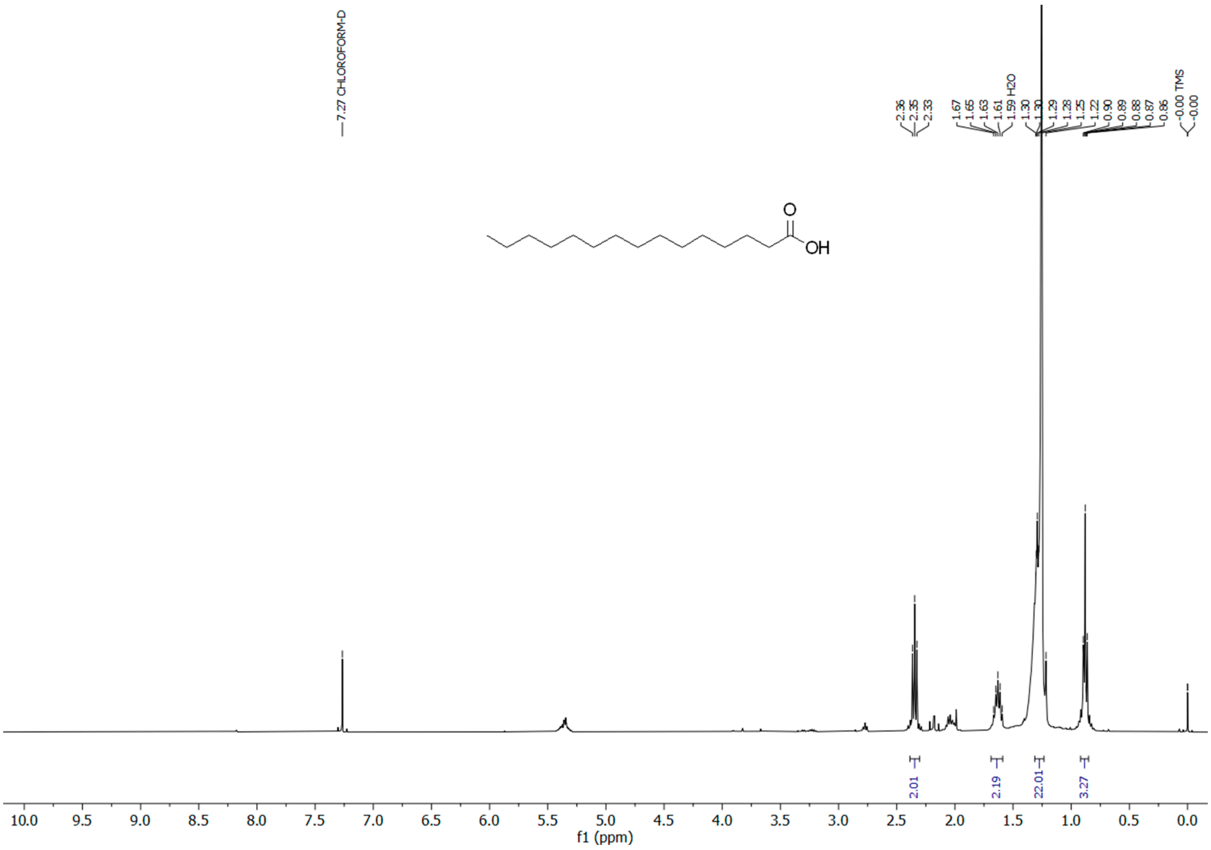

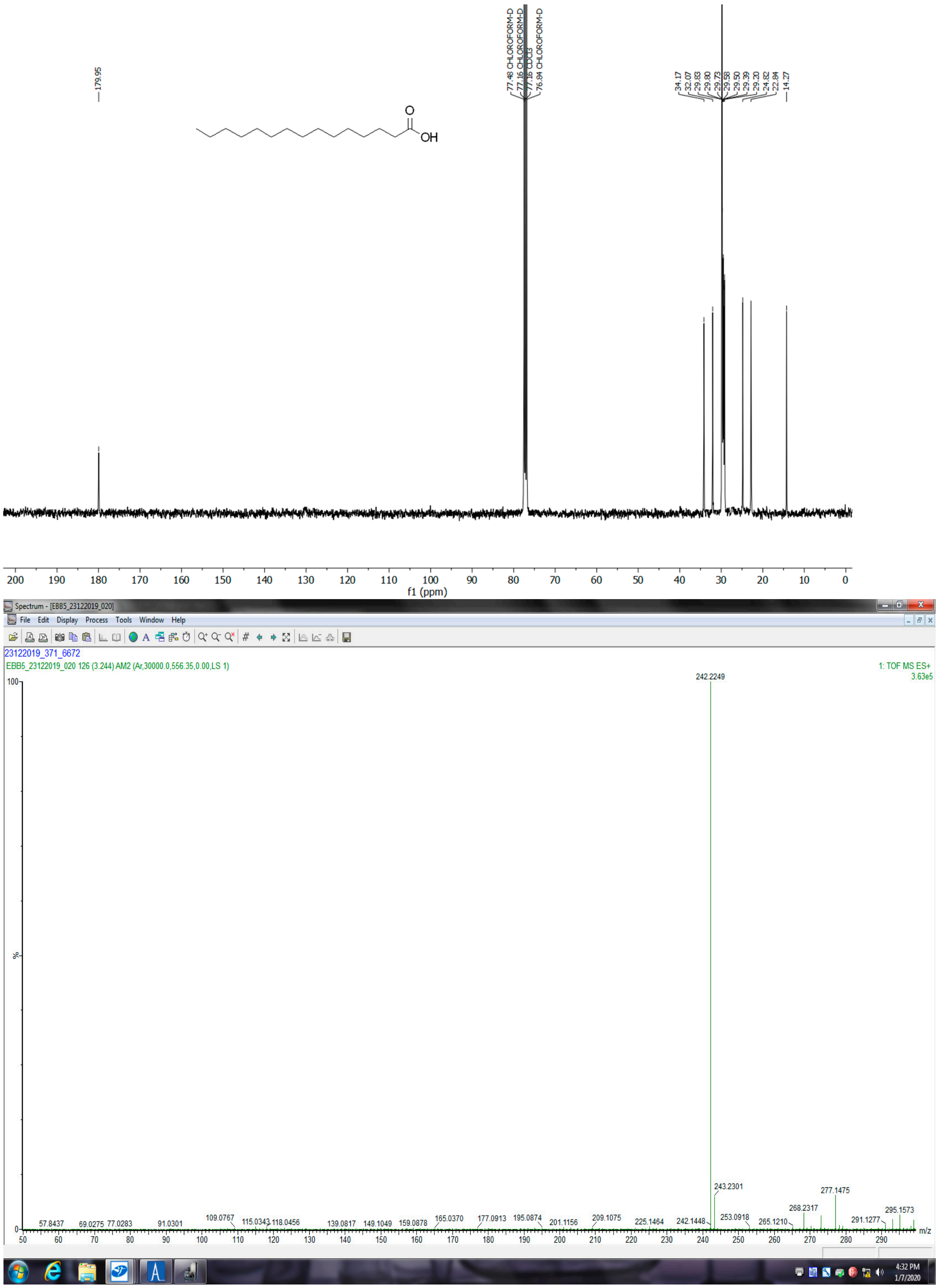

Figure **S1**. ^{1}H, ^{13}C-NMR and HRMS spectra for compound **1**

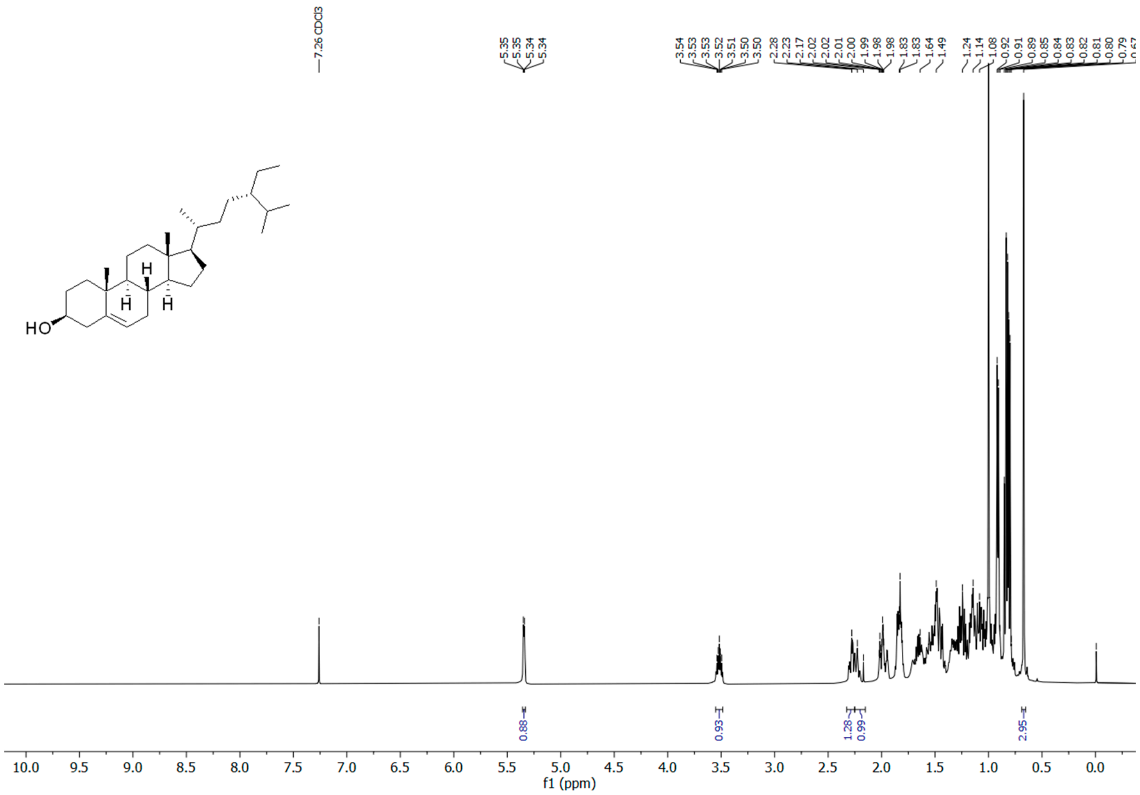

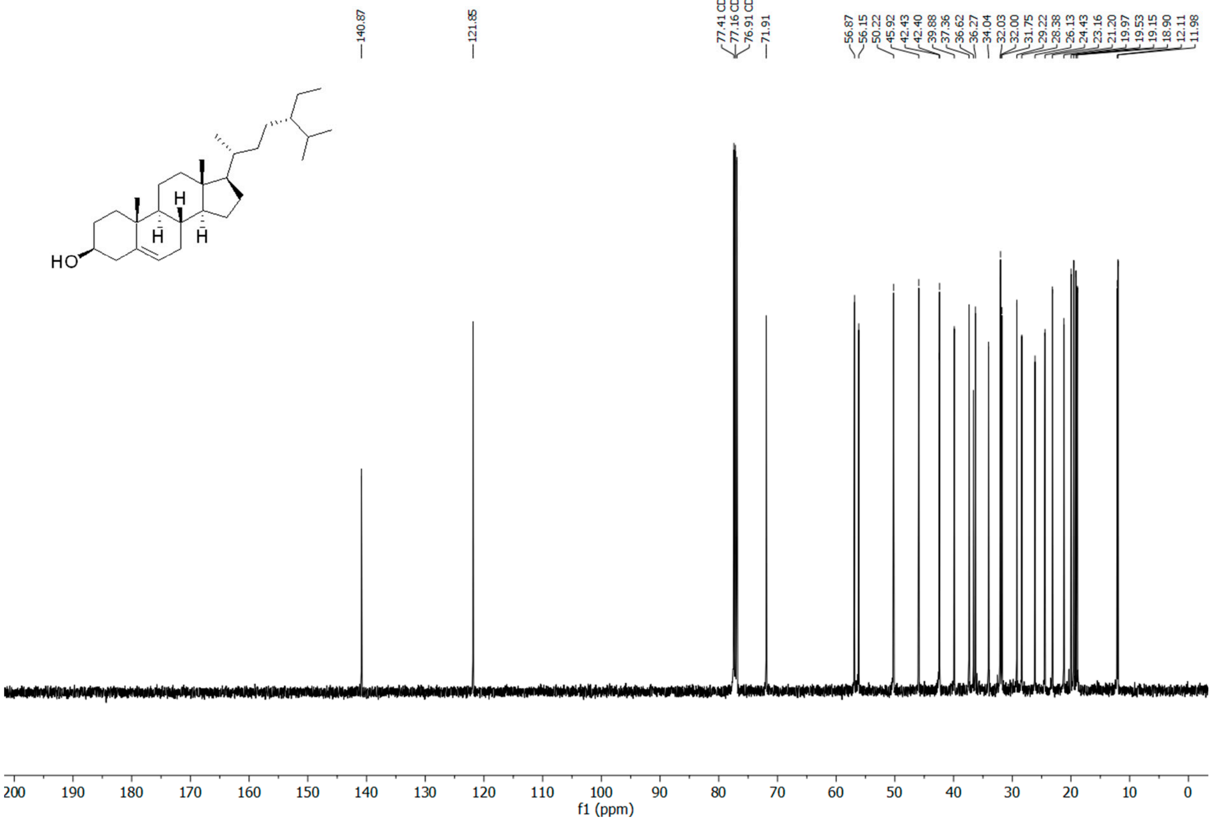

Figure **S2**. ^{1}H and ^{13}C-NMR spectra for compound **2**

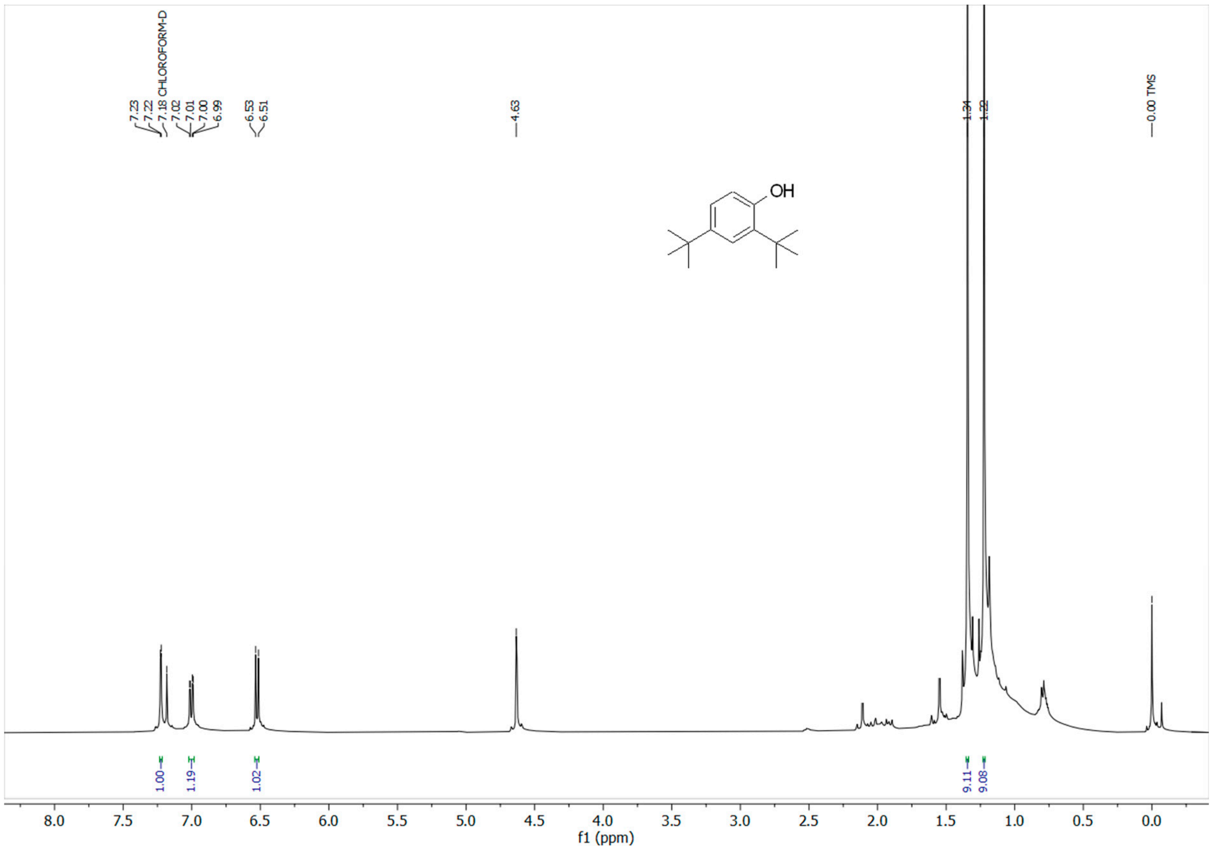
7.23
7.22
7.18 CHLOROFORM-D
7.02
7.01
7.00
6.99
6.53
6.51
4.63
1.34
1.22
0.00 TMS
OH
1.00
1.19
1.02
9.11
9.08
8.0
7.5
7.0
6.5
6.0
5.5
5.0
4.5
4.0
3.5
3.0
2.5
2.0
1.5
1.0
0.5
0.0
f1 (ppm)

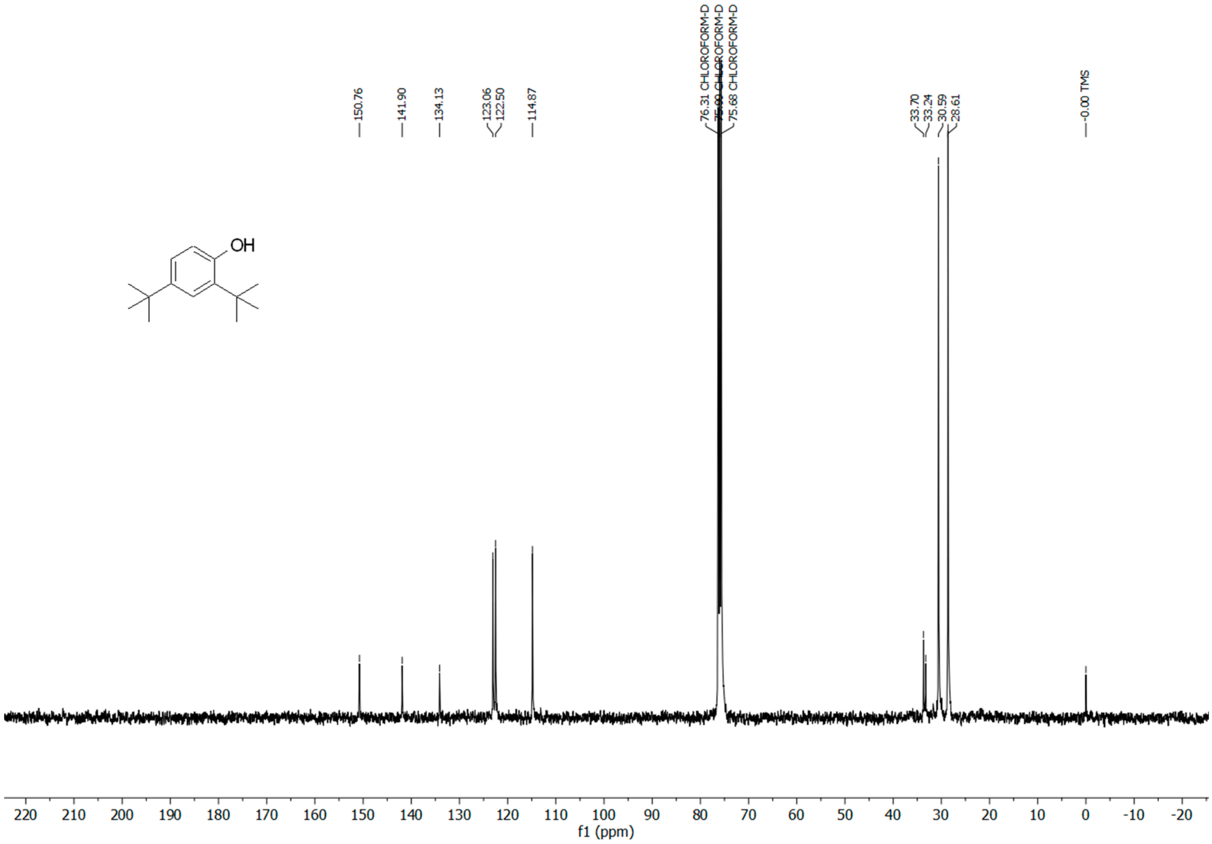
150.76
141.90
134.13
123.06
122.50
114.87
76.31 CHLOROFORM-D
CHLOROFORM-D
75.68 CHLOROFORM-D
33.70
33.24
30.59
28.61
0.00 TMS
OH
220
210
200
190
180
170
160
150
140
130
120
110
100
90
80
70
60
50
40
30
20
10
0
-10
-20
f1 (ppm)

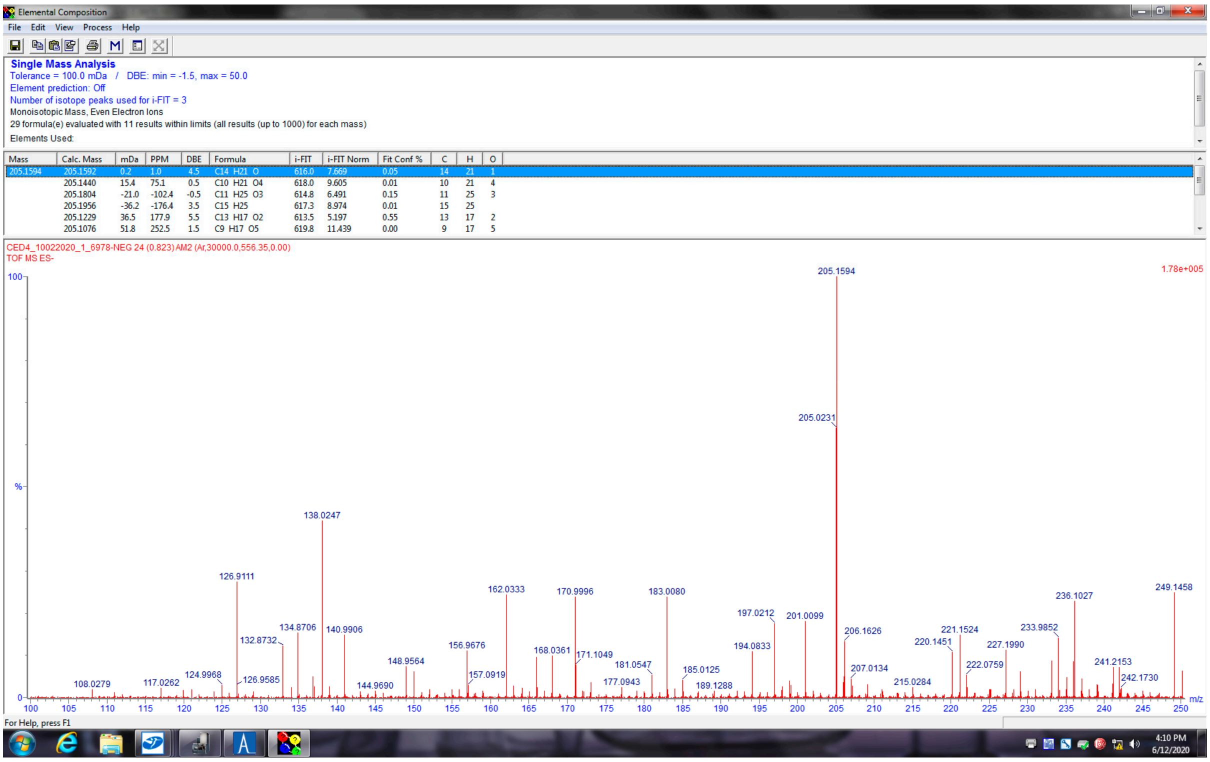

Figure **S3**. ^{1}H, ^{13}C-NMR and HRMS spectra for compound **3**

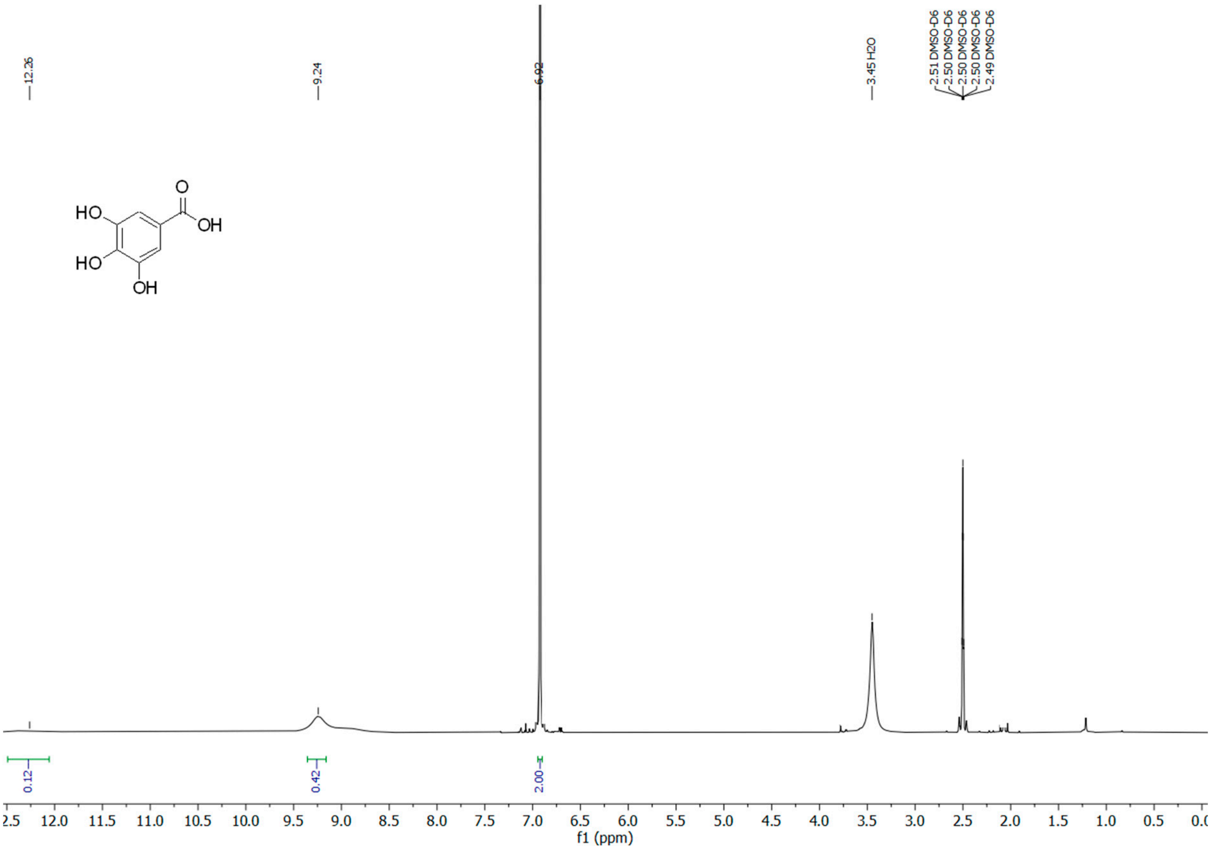

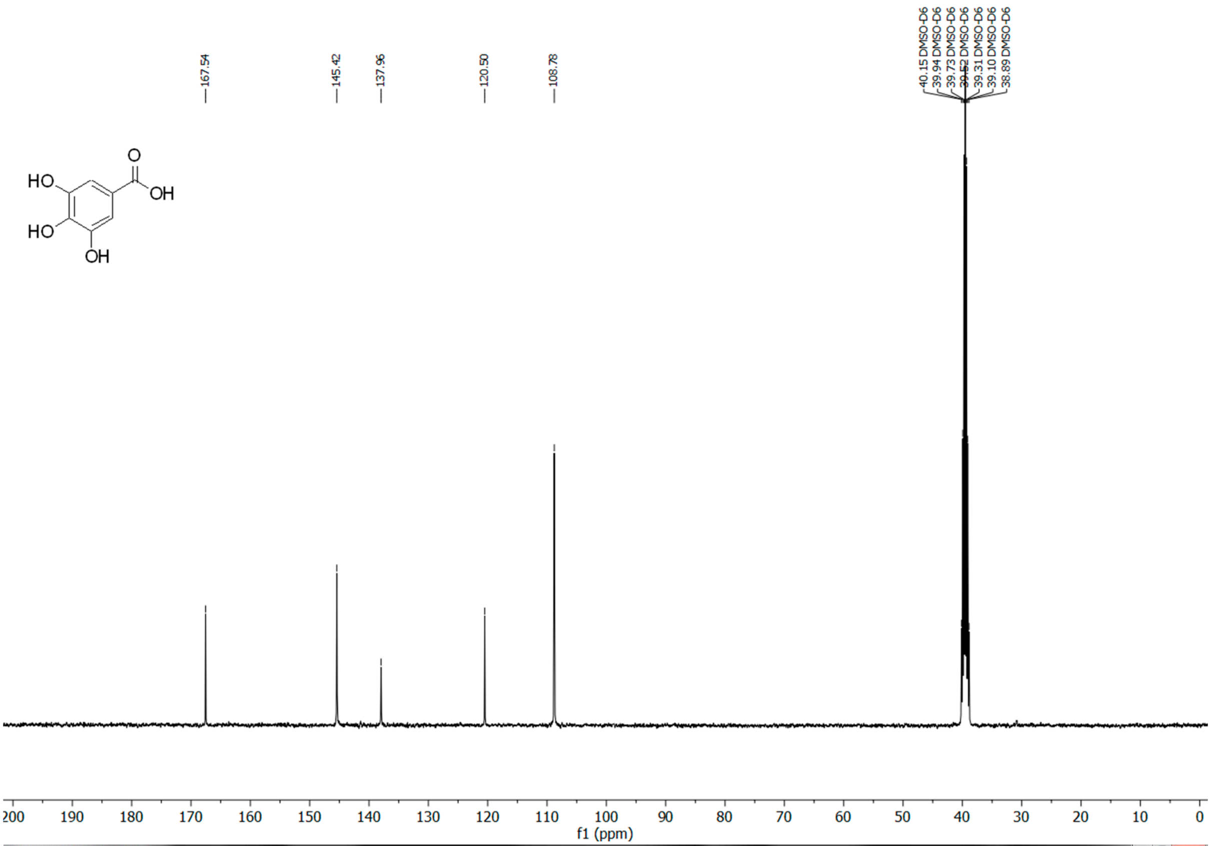

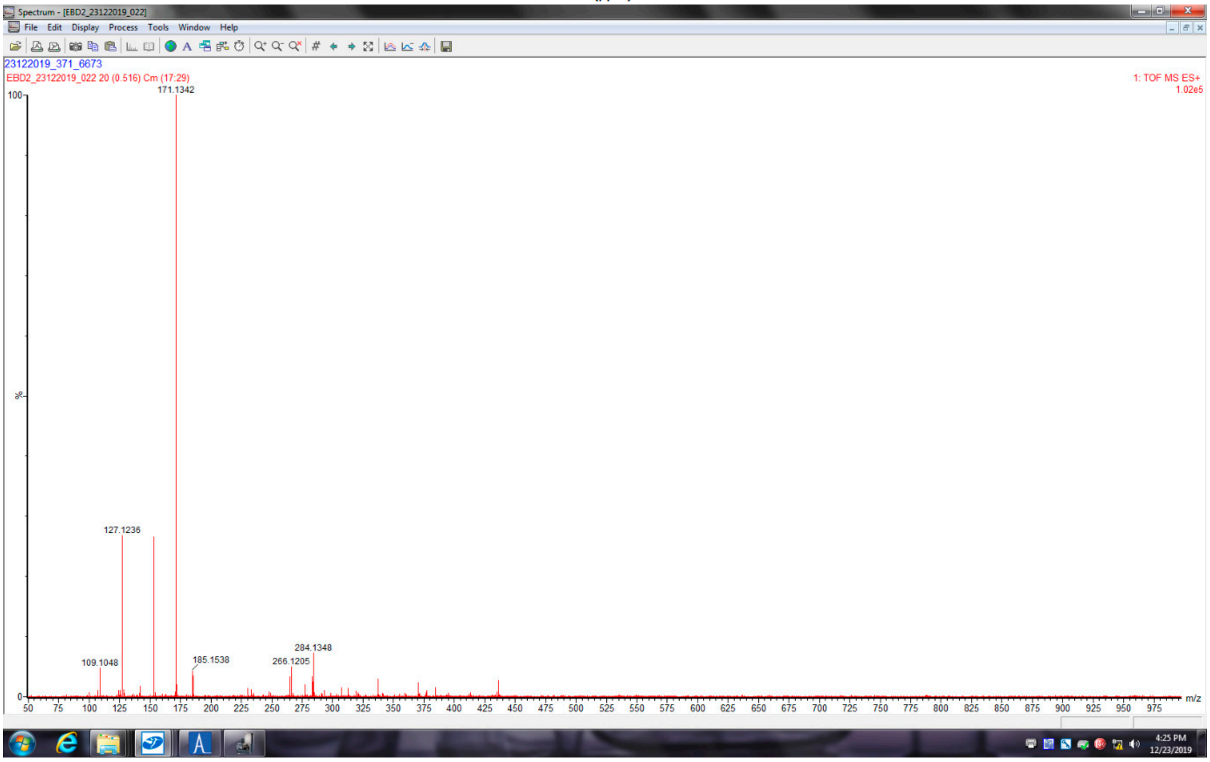

Figure **S4**. ^{1}H, ^{13}C-NMR and HRMS spectra for compound **4**

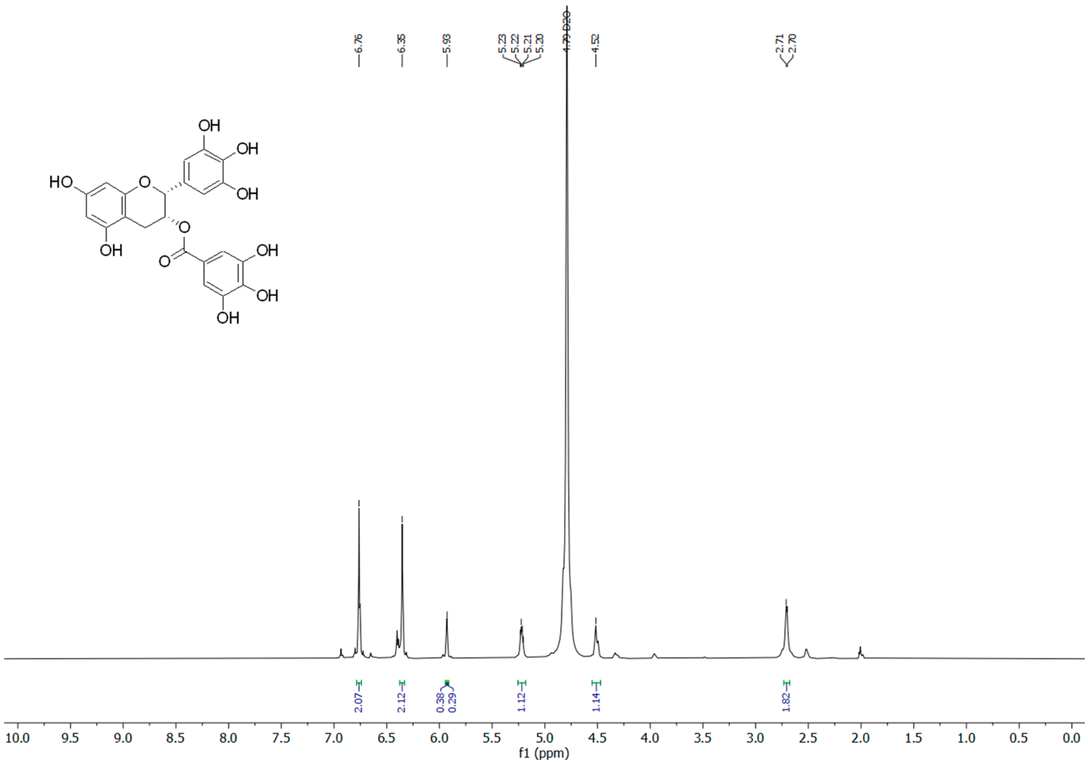

6.76
6.35
5.93
5.23
5.22
5.21
5.20
4.79 D2O
4.52
2.71
2.70
HO
O
OH
OH
OH
OH
O
O
OH
OH
OH
2.07
2.12
0.38
0.29
1.12
1.14
1.82
f1 (ppm)

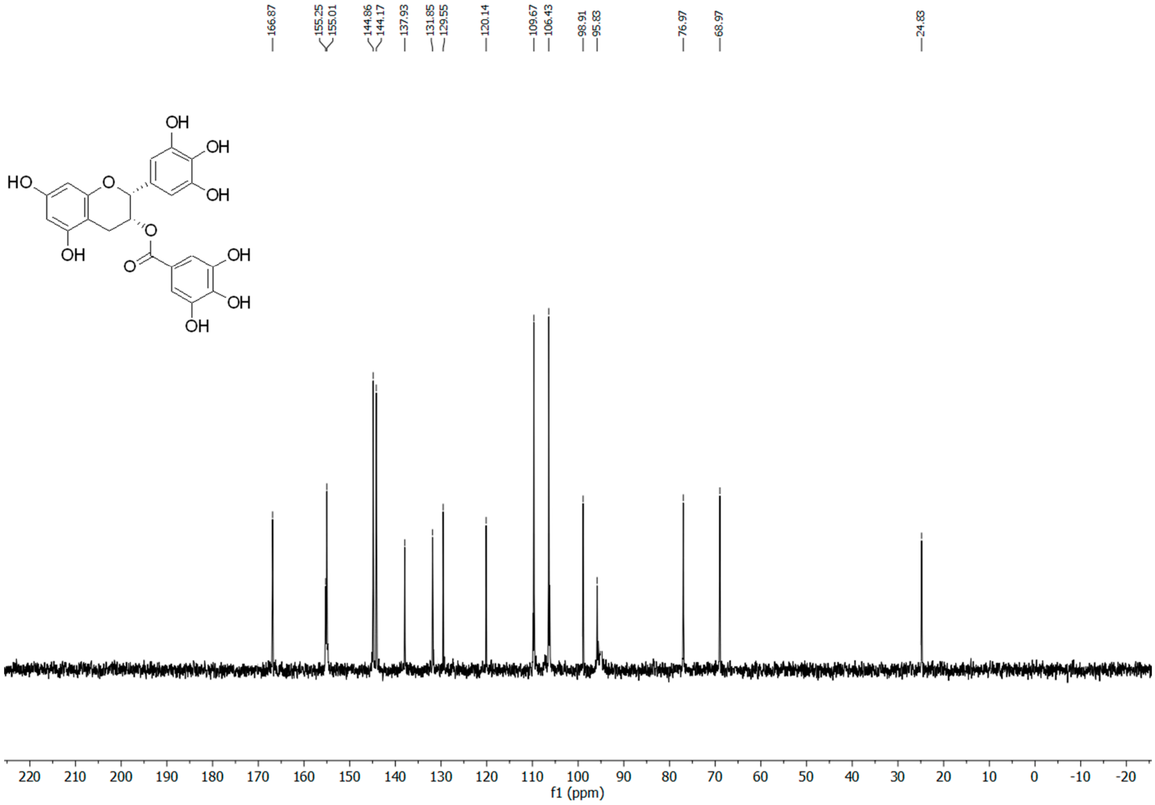

166.87
155.25
155.01
144.86
144.17
137.93
131.85
129.55
120.14
109.67
106.43
98.91
95.83
76.97
68.97
24.83
HO
O
OH
OH
OH
OH
O
O
OH
OH
OH
f1 (ppm)

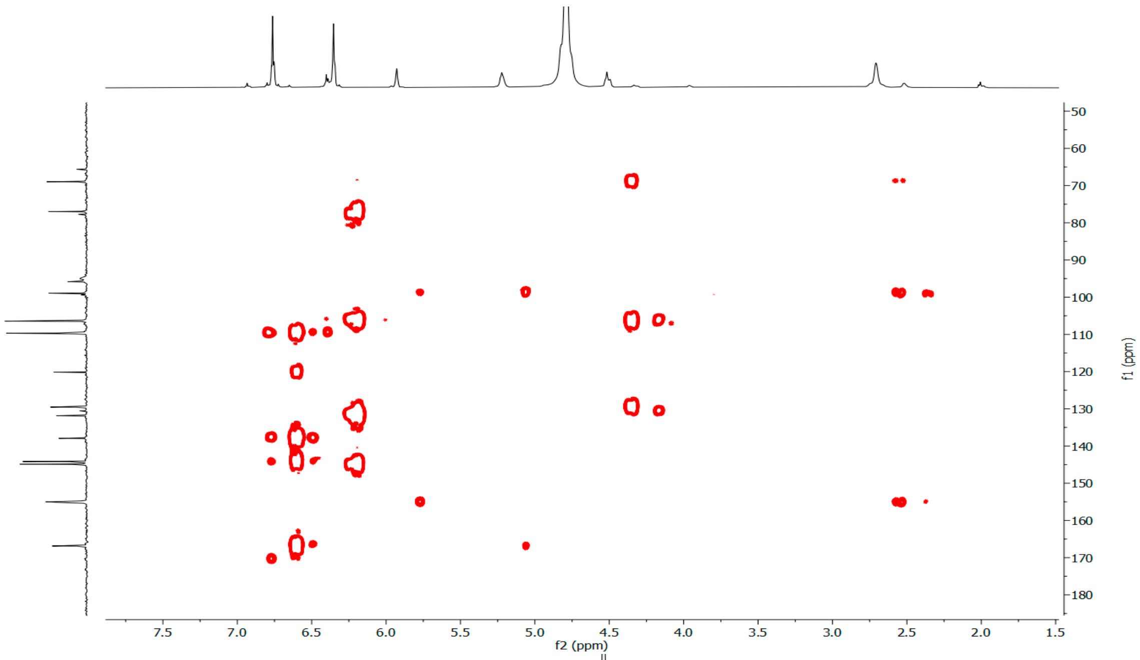

f2 (ppm)
f1 (ppm)

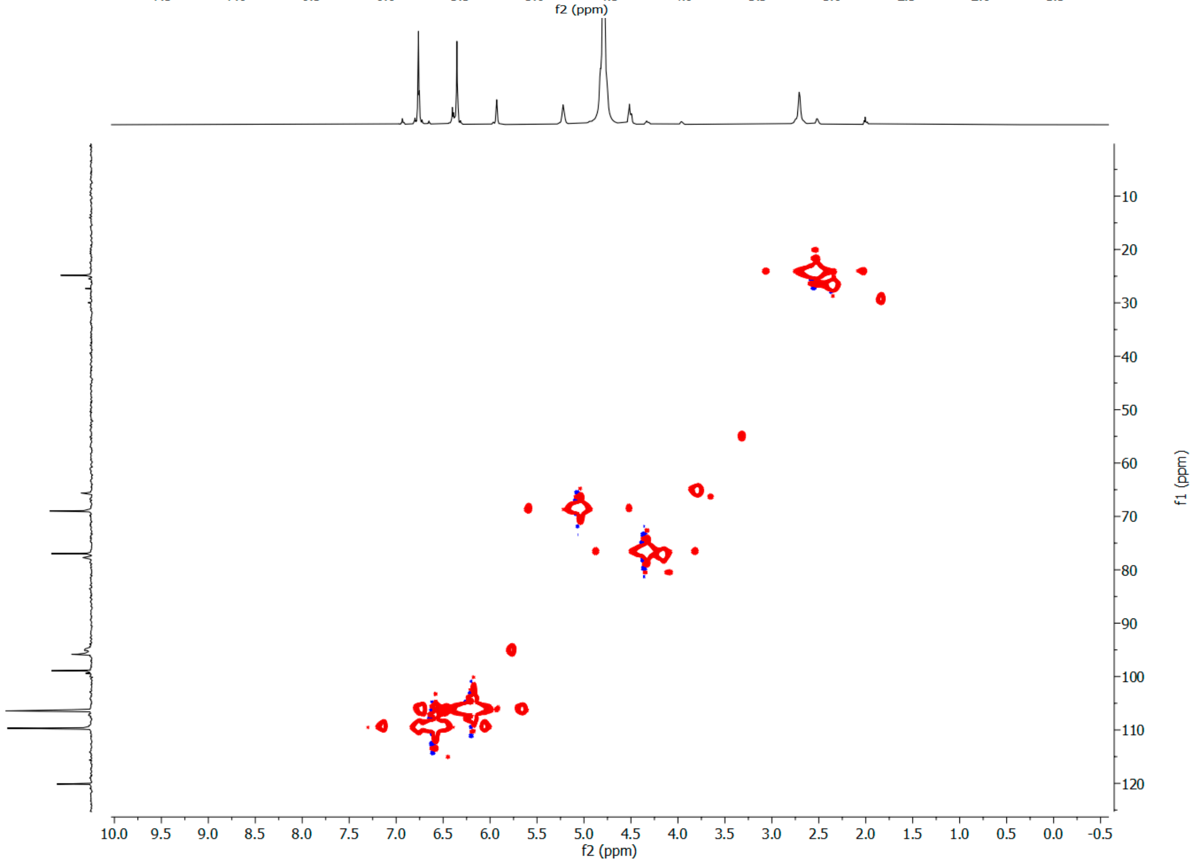

f2 (ppm)
f1 (ppm)

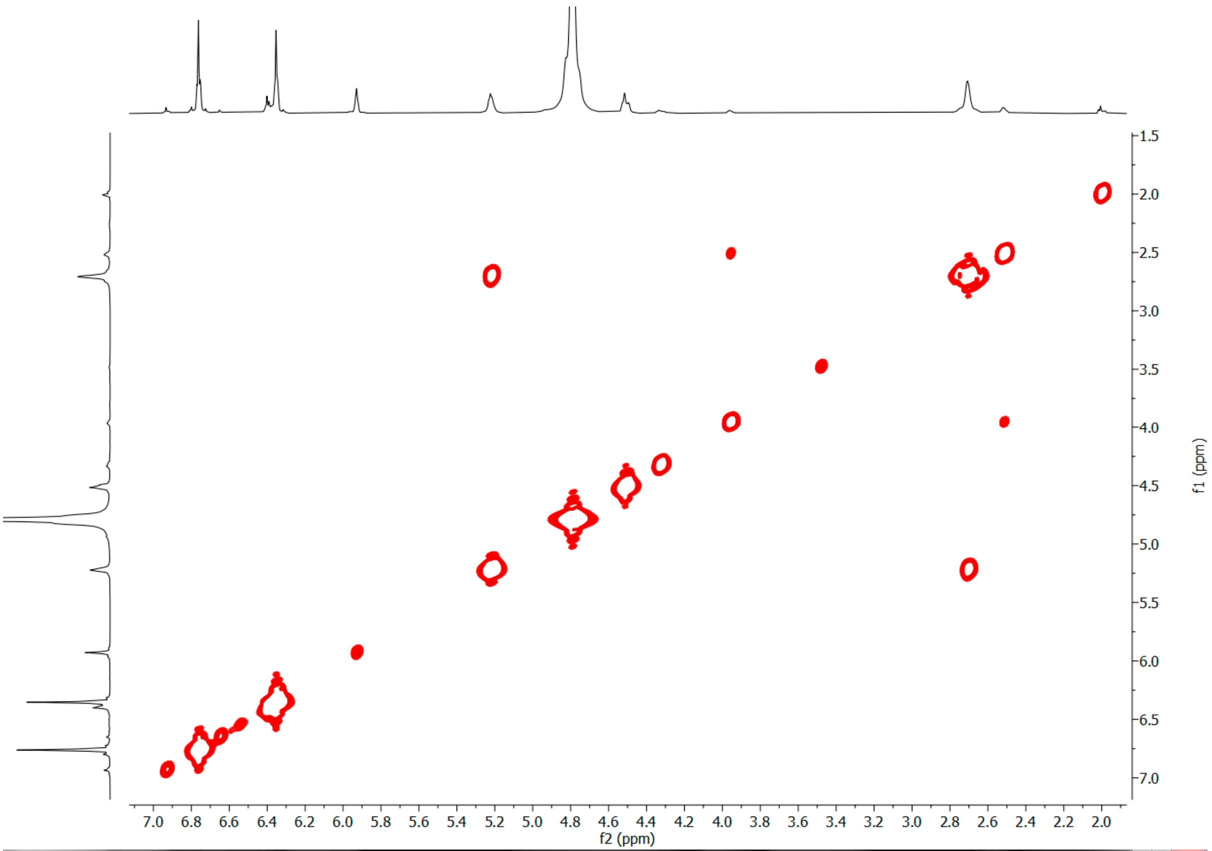

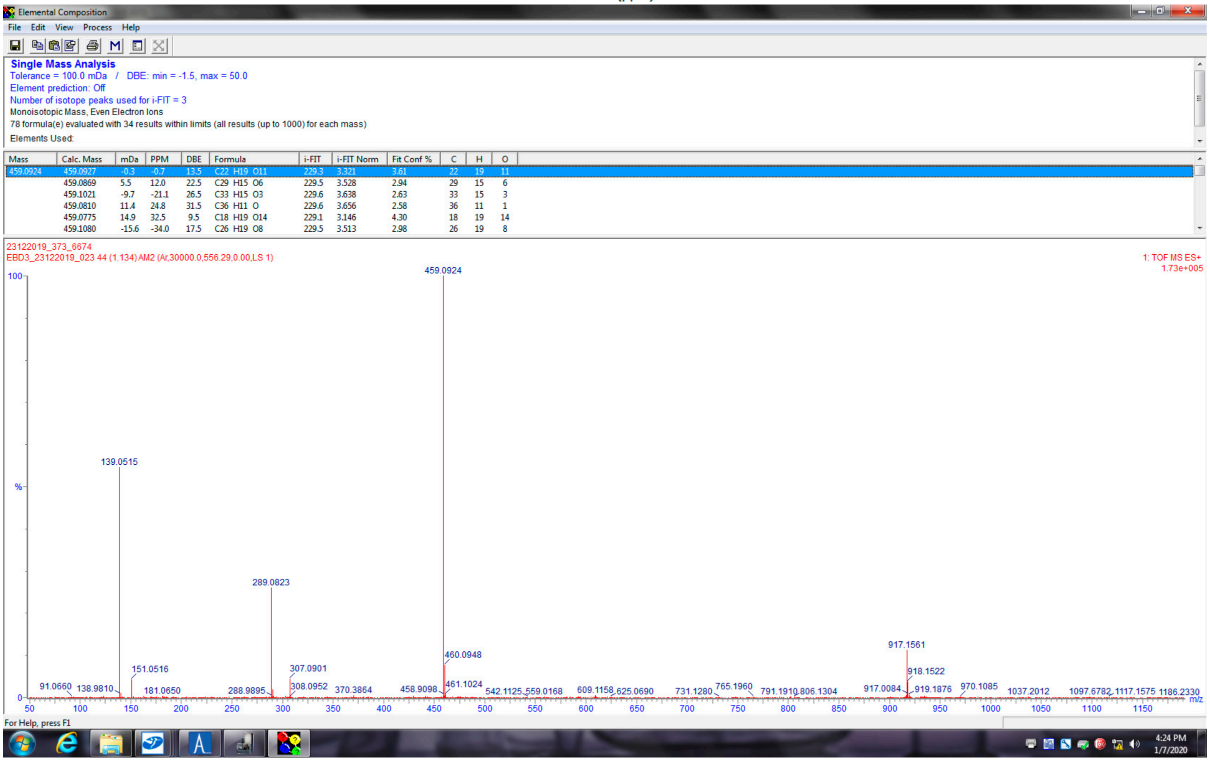

Figure **S5**. ^{1}H, ^{13}C-NMR, COSY, HSQC, HMBC, and HRMS spectra for compound **5**
